# Sociologists in public health: marginal observers or mainstream collaborators?

**DOI:** 10.1177/17579139231204245

**Published:** 2024-03-05

**Authors:** K Powell, NJ Fox, S Bhanbhro, A Chauhan, A Goldschmied Z, K Jackson, A Paton, S Salway

**Affiliations:** School of Health and Related Research, University of Sheffield, Regent Court, Sheffield, S1 4DA, UK; Department of Behavioural and Social Sciences, University of Huddersfield, Huddersfield, UK; College of Health, Wellbeing and Life Sciences, Sheffield Hallam University, Sheffield, UK; Institute of Psychiatry, Psychology & Neuroscience, King’s College London, London, UK; Department of Nursing and Midwifery, Sheffield Hallam University, Sheffield, UK; Population Health Sciences Institute, Newcastle University, Newcastle upon Tyne, UK; Centre for Health and Society, Aston University, Birmingham, B4 7ET, UK; Department of Sociological Studies, University of Sheffield, Sheffield, UK

*This article considers why sociology and public health do not collaborate more frequently and what sociologists might need to do to enhance their contributions to public health. It highlights a group of sociologists who have worked alongside public health practitioners that suggest ways to enhance sociology’s accessibility and use within public health, deriving from a workshop conducted in 2022.*

## Introduction

At first glance, sociology and public health should make for good partners. Both disciplines address the social, environmental, and community contexts of embodiment and well-being. Both are concerned with social inequality, social justice, and the politics of policy-making. Both are staffed by committed professionals who engage with the public, community leaders, and stakeholders to make a difference to people’s lives.

However, the marginal influence of sociology within UK public health became apparent during the pandemic^1^ in its role in UK Government scientific advisory groups. Sociological insights were missing, for instance, in responses to class, ethnic, and gender variations in infection and care-seeking.^2^ The congruity of the disciplines has been recognised in recent UK public health guidance^3–5^ which identifies a need to enhance public health’s collaborative work with sociologists. So why is it that sociology and public health do not collaborate more? And what might sociologists do to enhance their contributions to public health? Here, a group of sociologists suggest some solutions, deriving from a workshop conducted in 2022.

## Barriers to Collaboration Between Public Health and Sociology

Much of the failure of sociology to contribute more substantively to public health policy and practice derives from disciplinary boundaries.^6,7^ First, unlike psychologists and economists, ‘sociologists’ are predominantly employed within academic centres; physically distant from public health practitioners, activists, and policy-makers; and driven (by university managerial metrics) to target outputs in often inaccessible academic journals. Second, the evidence-based model of healthcare replicated in public health^8^ has devalued sociological knowledge often generated through qualitative methods and theoretical frameworks, prioritising instead meta-analysis of randomised controlled trials. A sociological perspective requires alternatives to established experimental methods for evaluating the impact of planned interventions in collective terms.

**Figure:**
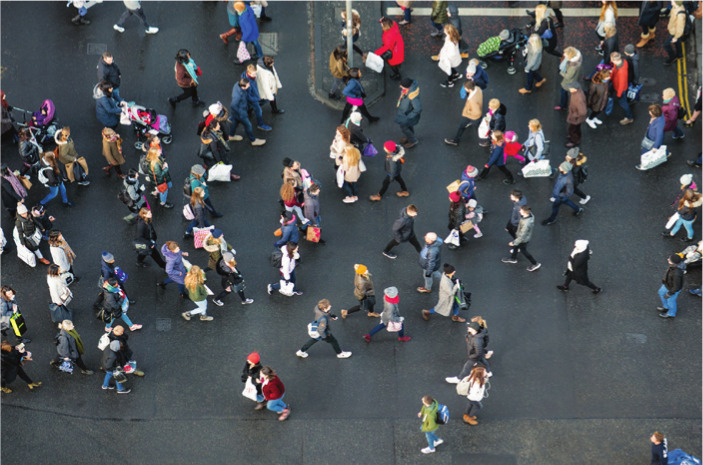


Finally, the disciplines can sometimes diverge in their worldviews, despite a focus on inequality. Public health models of social determinants of health^9,10^ can reify ‘the social’ as *contextual* risk factors for individual health outcomes^11^ overlooking diversities in what constitutes a ‘healthy life’ and the unequal distribution of power within societies. Social theories of power, however, are multiple and contested within the discipline^12^ and can seem abstract and inaccessible to public health practitioners and policy-makers concerned with the immediate practical challenges of health and social inequalities.

The move of public health into local government in the UK has opened more opportunities for collaboration, and from experience, we recognise that sociological concepts (such as ‘intersectionality’) and methods (such as focus groups) are frequently applied within public health without acknowledging their disciplinary origins. In the spirit of breaking down barriers, the rest of this commentary considers how sociologists have contributed to public health knowledge, before outlining four proposals to move this collaboration forward.

## Approaches that can Harness Sociology’s Contribution to Policy and Practice

Sociologists need to interrogate their conventional model of research. As Karvonen et al.^13^ suggest,

*This requires new forms of data production and more intense interaction with end users and stakeholders . . . This means stepping out of the traditional superiority position . . . into a position that is accountable and dialogical with the ‘publics’, whether lay people or other professionals.*

Where sociologists have made a difference, often they have found ways to locate themselves physically or embed themselves and their research activities within practice communities. In the UK, the University of Huddersfield has seconded sociologists to work at Kirklees District Council to develop tools to assess health inequalities and re-purpose impact assessments to enhance practice.^14^ Blake^15^ describes how a research assistant was embedded within a community organisation in a former UK coal mining community, deploying social theory to explore opportunities to improve food security. Beyond the UK, sociologists have worked with public health professionals and communities on community development issues. A multidisciplinary team of US academics and students from Purdue University worked with citizen groups in Hartford, Indiana, to generate evidence of heavy metal pollution and subsequently engaged with environmental regulators and local government to address this.^16^ Sociologists in Trondheim, Norway, established a sociology clinic in the shopping area of the city to offer sociological solutions to citizens’ issues. This in turn led to projects working with urban planners, commercial endeavours, and community bodies and citizens.^17^

## Four Proposals to Support Public Health/Sociology Collaborations

We suggest four interventions as ways to enhance sociology’s accessibility and use within public health.

It is essential to research the practical needs and priorities for people in both disciplines, and to document current examples of collaboration. The outreach projects described above pose major funding and human relations questions. An increasing emphasis on impact by research funders is influencing work in multidisciplinary teams that include a range of publics, but questions remain. What financial and governance models for embedding sociologists in non-academic settings might be developed? How are these sociologists to be mentored and supported if they are physically and culturally distanced from the scholarly community of sociologists? What is the career structure and what are the opportunities for advancement exist for sociologists working in non-academic settings or devoting their efforts to applied projects that may not generate scholarly outputs? In the US and some other nations, applied sociologists have been professionalised, with the development of graduate programmes and professional accreditation bodies such as the *Association for Applied and Clinical Sociology*. Evaluation of previous collaborations could reveal important enablers.

A second action is to explore in more detail how existing sociological concepts, tools, and methods are used in practice, and how these may be adapted or developed to meet the particular needs of public health. Co-development and piloting of toolkits could be facilitated by bodies such as the *Association of Directors of Public Health* and the British Sociological Association’s *Applied Sociology Group*.

The third intervention looks at how to raise the profile of sociology within public health and build alliances. A more robust, visible, and accessible public engagement is necessary to show how sociological approaches influence diverse public health projects, both through traditional academic dissemination and ‘public sociology’ initiatives such as blogs and podcasts. This can be enabled by collaborations between our professions to collate evidence-based case studies, public engagement projects, and impact studies.

Finally, work is needed to ensure that the next generations of both sociologists and public health professionals acknowledge the value of each other’s perspectives through both formal educational programmes and continuing professional development. This could include enhancing sociological input within public health degree courses; supporting undergraduate sociology curricula to include modules on applied research; using a range of public health case studies and creating placements in public health; and providing health practitioners with opportunities to co-work with sociologists during their training.

We offer this commentary as a first step and invite our public health and sociological colleagues to share our different competences, to improve the health of the public.
